# Development of a new equation and validation of earlier resting energy expenditure predicting equations in adults living in Tehran

**DOI:** 10.34172/hpp.42570

**Published:** 2024-07-29

**Authors:** Yahya Jalilpiran, Neda Azizi, Kimia Alipoor, Sanaz Mehranfar, Mojdeh Ebaditabar, Sakineh Shab-Bidar, Alireza Ostadrahimi, Kurosh Djafarian

**Affiliations:** ^1^Department of Clinical Nutrition, School of Nutritional Sciences and Dietetics, Tehran University of Medical Sciences (TUMS), Tehran, Iran; ^2^Department of Community Nutrition, School of Nutritional Sciences and Dietetics, Tehran University of Medical Sciences (TUMS), Tehran, Iran; ^3^Department of Nutrition, School of Public Health, Shahid Sadoughi University of Medical Sciences, Yazd, Iran; ^4^Nutrition Research Center, Tabriz University of Medical Sciences, Tabriz, Iran

**Keywords:** Cross-sectional studies, Indirect calorimetry, Resting energy expenditure

## Abstract

**Background:**

Predictive equations have been considered as a practical approach for estimating resting energy expenditure (REE) across multiple populations, but their accuracy for each community remains to be determined. Thus, the purposes of this study were to determine the validity of REE predictive equations and to develop a new REE predictive equation in adults living in Tehran.

**Methods:**

The study included 284 subjects (158 females) aged 18-60 years old from two cross-sectional studies conducted in Tehrani populations. Anthropometric measurements were assessed using standard protocols. REE was measured using indirect calorimetry (IC) and was estimated using preexisting equations. A new equation was also developed based on the REE from IC and variables such as age, sex, height, and weight. Measured REE was compared to new equation and preexisting predictive equations via correlation, linear regression, and Bland-Altman tests.

**Results:**

The new equation and the equations by Mifflin—St. Jeor, Livingston, Frankenfield, Nichols, Müller, and Ganpule demonstrated the best predictive value at a group level (mean percentage error=-2.2 to 2.4 %). At an individual level, the new equation and the equations by Mara, Frankenfield, Roza, Nikooyeh, and Harris & Benedict showed the greatest accuracies compared to measured REE (accuracy prediction=50-53%).

**Conclusion:**

This study highlights the importance of considering race when predicting REE. It also demonstrates that the newly developed equation is more appropriate in a clinical setting at group but not individual level. Thus, further research is needed to examine the new equation in an independent sample.

## Introduction

 Food deprivation, starvation, and malnutrition are challenging issues in many parts of the world particularly in Latin America, Africa, and Asia. On the other hand, people in some countries, such as New Zealand, some European countries, Australia, and America, are affected by overweight and obesity, and in some others are affected by both.^1^ The main mechanisms for the development of obesity and malnutrition are unclear, however, the evidence shows energy imbalances between energy intake and energy expenditure are linked to metabolic disorders.^2-5^

 Accurate estimation of total energy expenditure (TEE) is important for establishing dietary intake targets in weight and nutritional management of subjects to minimize the negative consequences of overfeeding and underfeeding.^6,7^ The TEE is made up of three major components: resting energy expenditure (REE), physical activity energy expenditure, and thermic effect of food.^8^ Among these, the REE is an important element in prescribing energy demands because it accounts for 60%-70% of daily energy expenditure in sedentary adults^8^ providing the foundation for achieving a desired degree of energy deficit. The gold standard for measurement of REE is indirect calorimetry (IC), but its complex nature, the costs of equipment, and the need for trained personnel make it impractical in clinical settings.^9^ So, it makes the use of equations very wise to estimate REE.^10^

 Over time, numerous equations have been developed for the estimation of REE. However, based on a review, the majority of these equations were developed in Western populations.^11^ In addition, several studies revealed that the most commonly used equations for the prediction of REE such as Harris-Benedict,^12^ Schofield,^13^ FAO/WHO/UNU,^14^ and Mifflin et al^15^ over-estimated the REE in Asians.^16-20^ It appears that an individual’s race is a crucial issue that has been shown to play an important role in the variation in energy expenditure.^10,21^ Also, as far as we know there is no study investigating the validity of all available formulas in predicting REE in adults in Iran. Therefore, the purposes of this study were to determine the accuracy and validity of REE predictive equations and to develop a new REE predictive equation in a sample of Tehranian adults.

## Material and Methods

###  Study design and participants 

 Baseline data of 284 healthy Tehranian adults from two cross-sectional studies were pooled and were served as the basis for this analysis. Accordingly, we pooled the data of those studies which conducted under the consideration of Tehran university of medical sciences and had the same criteria. Information about the first study was reported previously.^22^ In the second study which included 100 participants we aimed to enter subjects with the same criteria as the first study (grant number: 99-3-212-51375). In both studies, various marketing methods were utilized to inform subjects about the research, such as flyers, ads, and in-house sessions. Overall, the participants from both studies who have had the following inclusion criteria participated in this analysis. (1) healthy adults aged 18-60 years old, (2) subjects without apparent alcohol or drug abuse, and (3) participants without any history of cardiovascular diseases, heart failure, thyroid dysfunction, malignant diseases, hepatic or renal diseases, severe asthma, and pulmonary diseases. Additionally, individuals who were on special dietary regimens, who were professional athletes, who took special drugs or supplements, or who were pregnant or lactating women were not included in the analysis.

###  Anthropometric measurements

 In light-clothes and shoeless conditions, individuals’ weights were measured using a digital scale to the nearest 0.1 kg. Height was measured using a wall-mounted, tape measure without shoes to the nearest 0.5 cm. Then, body mass index (BMI) was calculated by the following equation: BMI = bodyweight (kg)/height^2^(m). Waist circumference (WC) was measured at the narrowest point between the inferior rib and iliac crest over light clothing, without any pressure on the body and recorded to the nearest 0.5 cm. Hip circumference (HC) was measured at the horizontal level around the buttocks that yielded the maximum measurement.^23^

###  Indirect calorimetry 

 REE was measured using the IC method (Cortex Metalyser 3B, Leipzig, Germany) at room temperature (25 ± 2 ^°^C) while the subject was in a supine position with a face mask and light clothing. Each participant entered in REE-IC measurement if (*a*) she/he was on an at least 12-hour fasting condition, (*b*) did not use alcohol or caffeinated foods or supplements at least 4 hours before the test, (*c*) did not smoke at least 2 hours before the test, and (*d*) was not perform aerobic and anaerobic exercises at least 2 and 14 hours before the test, respectively. For females, REE-IC measurements weren’t performed when the subjects were in the luteal phase.^24^ Participants were also adapted to the test environment for about 30 minutes before the experiment. The experiment was conducted over 30 minutes, however, the first and the last 5 minutes were not included and were discarded.

###  REE predictive equations

 The PubMed database was searched 1959 up to February 10, 2023 for retrieving relevant published studies using the following search terms: [(‘’energy metabolism’’ or ‘‘basal metabolism’’ or ‘‘basal metabolic’’ or ‘‘resting metabolic rate” or ‘‘resting metabolic expenditure” or ‘’calorimetry, indirect’’) AND (‘‘measure*‘’ or ‘‘predict*’’ or ‘‘estimat*’’ or ‘‘equation*’’ or ‘‘formula*’’ or “valid*” OR “accurac*” OR “precis*”)]. We also searched the reference list of included studies to find the possible missed articles. For analysis, a study has been included if (*a*) performed on adults, (*b*) had sample sizes greater than 50 subjects, and (*c*) did not perform in special populations (e.g., only obese, only women, athletes, or patients). Also, equations that depended on variables other than age, sex, height, weight, and BMI were excluded, as these intricate measurements were impractical for everyday use in clinical settings.

###  Statistical analysis

 Continuous and categorical variables were expressed as the means ± standard deviations and frequencies (percentages), respectively. To develop a new equation, the whole sample (N = 284) was randomly separated into development (n = 142) and validation (n = 142) groups. The mean values of demographic, anthropometric measurements, and REEs (predicted and measured) in two groups were compared using student’s *t* test. The distribution of categorical variables in the two groups was compared using the Chi-square test. In the development group, a stepwise multivariable linear regression was performed to develop the new equation, using REE-IC as the dependent variable and age, sex, weight, and height variables as the independent variables. Accordingly, an REE-PE for each subject was calculated to the corresponding validation group. Student’s paired t-test was used to assess the difference between REE-IC and REE-PEs. Pearson’s correlation coefficients (R) were used to assess the relations between REE-IC and REE-PEs. Linear regression analysis was also performed to compute R^2^ and root mean square prediction error (RMSE) of each predictive REE equation. To assess the agreement between REE-IC and REE-PEs, Bland-Altman’s method was used by plotting the differences between the REE-PEs and REE-IC against the average values of them.^25^ The accuracy rate was calculated as the percentage of subjects whose REE-PE was within ± 10% of the REE-IC, a level commonly used to determine the accuracy of a PE at individual level.^26^ Mean percentage error was used to check the agreement between the REE-IC and REE-PEs at group level. A two-tailed *P* value < 0.05 was considered to be statistically significant. Statistical analyses were performed using IBM SPSS Statistics (version 24, IBM Corp., Armonk, USA) and STATA software (version 14).

## Results

 The study included 284 subjects (126 males, 158 females) aged 18-60 years old from two cross-sectional studies conducted in Tehranian populations. As shown, between-group comparisons didn’t indicate any significant difference between the development and validation groups in terms of general characteristics, anthropometric measurements, and REE-IC **(**Table 1).

 In the development group, the new equation was calculated by including weight, height, sex, and age as major predictors of REE as follows: REE = 8.957 × weight + 280.613 × sex (male = 1, female = 0) - 7.795 × age + 1039.837 (R = 0.64, R2 = 0.42, adjusted R2 = 0.40, *P* < 0.001). During the validation phase, the new equation was tested for predictability, resulting in an R2 value of 0.54 (*P* < 0.001), with a prediction mean bias of -14 kcal/day. Based on the literature review of the equations by Harris and Benedict,^12^ Roza and Shizgal,^27^ FAO.WHO.UNU,^28^ Schofield,^13^ Owen et al,^29^ Mifflin et al,^15^ De Lorenzo et al,^30^ Müller et al,^31^ Livingston and Kohlstadt,^32^ Ganpule et al,^33^ Korth et al,^34^ Frankenfield et al,^10^ de la Cruz Marcos et al,^35^ Fairoosa et al,^36^ Marra et al,^37^ Nikooyeh et al,^38^ and Nichols et al^39^ had the criteria to use in this study (Table 2).

Table 3 shows the precision of REE-PE by the new equation and previously published equations compared to the REE-IC in the validation group. Accordingly, REE-PE from previously published equations and the new equation showed similar results regarding correlations with REE-IC (Pearson’s r = 0.72–0.75; all *P* < 0.001), except for Müller et al^31^ equation, where the correlation was less strong (Pearson’s r = 0.68; *P* < 0.001) with the highest RMSE. However, there was a significant difference between the mean of REE obtained from Roza and Shizgal,^27^ FAO.WHO.UNU,^28^ Owen et al,^29^ De Lorenzo et al,^30^ Ganpule et al,^33^ Korth et al,^34^ Frankenfield et al (with height),^10^ de la Cruz Marcos et al,^35^ Fairoosa et al,^36^ and Nichols et al^39^ equations and REE-IC (for other equations the results were not significant). Among all predicted equations compared in this study, the equations by Harris & Benedict,^12^ Roza and Shizgal,^27^ Nikooyeh et al,^38^ Mara et al,^37^ and Frankenfield et al^10^ showed the highest accuracy at the individual level (accurate prediction at 50%-53% of the sample).

**Figure 1:**
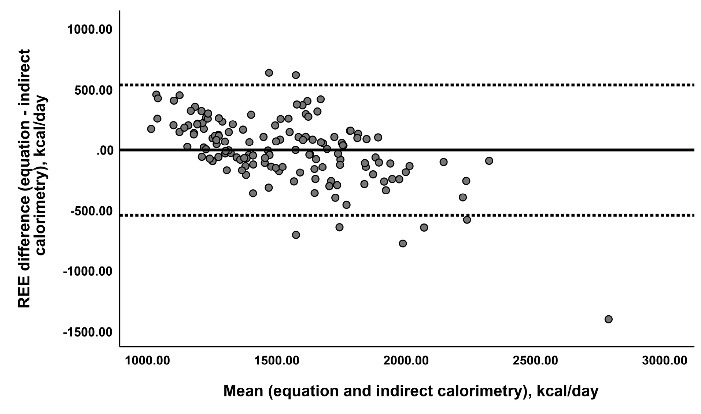
Bland–Altman plot for new resting energy expenditure (REE) predictive equation. The vertical line shows REE predicted by new equation minus REE measured by indirect calorimetry (IC) and the horizontal line shows the average of REE predicted by new equation and REE measured by IC. The solid horizental midline shows the mean difference between REE predicted by new equation and REE measured by IC method. The uper and lower dashed lines show the 95 % limits of agreement.

Table 4 indicates probable mean percentage error at the group level between REE-PEs and REE-IC and agreement between the methods via Bland-Altman analysis (also the result for the new equation is presented in Figure 1). Accordingly, the equations by Mifflin et al,^15^ Müller et al,^31^ Livingston and Kohlstadt,^32^ Ganpule et al,^33^ Frankenfield et al (without height),^10^ and Nichols et al^39^ showed the lowest mean percentage error compared to the REE-IC (lower than 3%). Nevertheless, the results showed proportional bias in all equations, suggesting that the difference between estimated and REE-IC increased as average REE increased. Regarding the new equation, it showed relatively acceptable results compared to other equations. As shown in Table 3, it did not differ from REE measured by IC, had R^2^ = 0.54 and RAMSE = 272.31, and a prediction accuracy = 53%. It also showed relatively high accuracy at the group level (mean percentage error of 2.4%, Table 4). However, it presented a proportional bias, analogous to other previously published equations (rho = -0.59, *P* < 0.001).

**Table 1:** General characteristics and anthropometric measurements of all participants and between development and validation groups

**Variables**	**Total (N=284)**	**Development group (n=142)**	**Validation group (n=142)**	***P*** ** value**
Age (year)	33.35 ± 10.44	32.83 ± 10.78	33.87 ± 10.11	0.40
Females (%)	158 (55.6)	74 (46.8)	84 (53.1)	0.23
Weight (kg)	71.93 ± 16.36	73.09 ± 15.94	70.76 ± 16.74	0.23
Height (cm)	168.27 ± 9.50	168.84 ± 9.19	167.70 ± 9.79	0.31
Body mass index (kg/m^2^)	25.32 ± 4.98	25.56 ± 4.87	25.08 ± 5.09	0.42
Waist circumference (cm)	87.43 ± 12.86	88.12 ± 13.24	86.74 ± 12.53	0.37
Hip circumference (cm)	99.12 ± 8.88	99.73 ± 8.40	98.51 ± 9.32	0.24
Resting energy expenditure (kcal)	1555.66 ± 383.57	1573.08 ± 366.30	1538.25 ± 400.65	0.44
Respiratory quotient	0.89 ± 0.07	0.89 ± 0.07	0.88 ± 0.06	0.13

Data are presented as mean ± standard deviation or Number (%). In the development group, the new equation was calculated by including weight, height, sex, and age as major predictors. The new equation was tested for predictability in the validation group. Independent samples t test and chi-square test were used for comparison of quantitative and qualitative variables between the two groups.

**Table 2:** Resting energy expenditure predictive equations included in this study

**Reference**	**Participants**	**REE predictive equations**
Harris and Benedict^12^	N = 239 (136M; 103F), age 21-70 y, 25-124.9 kg, 150-200 cm	M: WT*13.7516 + HT*5.0033–AGE*6.755 + 66.473 F: WT*9.5634 + HT*1.8496-AGE*4.6756 + 655.0955
Roza and Shizgal^27^	N = 337 (168M; 169F), age 21-70 years, 25-124.9 kg, 150-200 cm	M: 13.397*WT + 4.799*HT–5.677*AGE + 88.362 F: 9.247*WT + 3.098*HT–4.33*AGE + 477.593
FAO.WHO.UNU^28^	Equation based on Schofield^13^; database extended to 11 000 subjects	M: 18-30y: 15.3*WT + 679 30-60y: 11.6*WT + 879 F: 18-30y: 14.7*WT + 496 30-60y: 8.7*WT + 829 M: 18-30y: 15.4 × WT + 0.27 × HT + 717 30-60y: 11.3 × WT + 0.16 × HT + 901 F: 18-30y: 13.3 × WT + 3.34 × HT + 35 30-60y: 8.7 × WT - 0.25 × HT + 865
Schofield^13^	N = 7173, N = 4814 > 18 y, BMI 21–24 N = 3388 Italians (47%), N = 615 tropical residents, N = 322 Indian 114 published studies, N = 7173 subjects (11 000 values, including group mean values); most European and North American subjects	M: 18-30y: 15.057 × WT + 692.2 30-60y: 11.472 × WT + 873.1 F: 18-30y: 14.818 × WT + 486.6 30-60y: 8.126 × WT + 845.6
Owen et al^29^	N = 104 (60 M; 44 F), age 18–82 y, 60-171 kg (M) 43-153 kg (F), BMI 18–50	M: WT*10.2 + 879 F: WT*7.18 + 795
Mifflin et al^15^	N = 498 (251 M; 248 F), N = 264 normal weight (129 M; 135 F), N = 234 individuals with obesity (122 M; 112 F), age 19–78 y, BMI 17–42	M: [10 × WT (kg)] + [6.25 × HT (cm)] – [5 × age (y) + 5] F: [10 × WT (kg)] + [6.25 × HT (cm)] – [5 × age (y) – 161]
De Lorenzo et al^30^	N = 320 (127 M; 193 F), age 18–59 y, BMI 17–40	M: (53.284*WT + 20.957*HTCM–23.859*AGE + 487)/4.184 F: (46.322*WT + 15.744*HTCM–16.66*AGE + 944)/4.184
Müller et al^31^	N = 2528 (1027 M; 1501 F), 5–80 y, BMI > 25	BMI < 18.5: 0.07122 × WT (kg)-0.02149 × age (y) + 0.82 × sex + 0.731 BMI ˃ 18 ≤ 25: 0.02219 × WT (kg) + 0.02118 × HT (cm) + 0.884 × sex-0.01191 × age (y) + 1.233 BMI ˃ 25 ≤ 30: 0.04507 × WT (kg) + 1.006 × sex - 0.01553 × age (y) + 3.407 BMI˃30: 0.05*WT-0.01586*AGE + 1.103*SEX + 2.924
Livingston and Kohlstadt^32^	N = 655 (299 M; 356 F), age 18–95 y, 33– 278 kg	M: 293*WT^0.433^– 5.92*AGE F: 248*WT^0.4356^–5.09*AGE
Ganpule et al^33^	N = 137, 71 M and 66 F; age ˃20 years	Men: (48.1 × WT + 23.4 × HT -13.8 × age - 547.3(male = 0) - 423.5)/4.186 Women: (48.1 × WT + 23.4 × HT-13.8 × age - 547.3(female = 1) - 423.5)/4.186
Korth et al^34^	N = 104 (50 M; 54 F), age 21–68 y, BMI 18-41	(41.5*WT + 35.0*HTCM + 1107.4*SEX (male 1; female 0)-19.1*AGE-1731.2)/4.184
Frankenfield et al^10^	N = 337 (94 M, 243 F), age > 18 y, age 18-85	BMI ≥ 30: WT*10−AGE*5 + SEX*274 + 865 BMI < 30: WT*11-AGE*6 + SEX*230 + 838 BMI ≥ 30: WT*10 + HTCM*3−AGE*5 + SEX*244 + 440 BMI < 30: WT*10 + HTCM*3−AGE*5 + SEX*207 + 454 Sex = (male 1; female 0)
de la Cruz Marcos et al^35^	N = 134 (67 M; 67 F), age 19-65 y	1376,4–308*SEX (male 0; female 1) + 11,1*WT–8*AGE
Nikooyeh et al^38^	N = 252 (121 M, 131 F), age 18-60, mean BMI = 27.2	M: 18-30y: 8.4*HT + 5*WT + 27.5*Age- 869.7 31-60y: 7.8*HT + 12.5*WT-5.64*Age- 349.9 F: 18-30y: 8.4*HT + 5*WT + 27.5*Age- 979.7 31-60y: 7.8*HT + 12.5*WT-5.64*Age- 455.4
Fairoosa et al^36^	N = 57 (27 M; 30 F), age 19-60	284.5 + (13.2 x WT) + (133.0 x sex) (male 1; female 0)
Marra et al^37^	N = 2483, age ˃18 year, BMI = 18.5.30 160 F and 66 M	70.4 + (12.1 x WT) + (3.83* HT) + (139*sex (male 1; female 0))- (1.82*age
Nichols et al^39^	N = 400 (148 M;252 F), age 20-65	295.92 + 171.29*sex (male 1; female 0) – 5.89*age + 10.52*WT (kg) + 3.30*HT (cm)
New equation	N = 284 (126 M, 158 F) (development group = 142, validation group = 142), age 18-60	8.957*WT + 280.613*SEX-7.795*Age + 1039.837 (the formula was obtained in the development group)

Abbreviations: M, male; F, female; y, years of age; kg, kilograms; cm, centimeters; BMI, body mass index; WT, weight in kg; HT, height in centimeters; REE, resting energy expenditure.

**Table 3:** Precision of REE-PE by the different equations compared to the REE-IC in the validation group (n = 142)

**REE predictive equation**	**REE, kcal/day**	***P*** **value** ^a^	**Pearson’s r**	**R** ^ 2 ^	**Adjusted R** ^ 2 ^	**RAMSE**	**Accurate predictions (% subjects)**	**Under and over predictions (% subjects)**
Measured REE (Indirect calorimetry)	1538.25 ± 400.65	-	-	-	-	-	-	-
Harris and Benedict^12^	1577.99 ± 275.29	0.08	0.74	0.544	0.541	271.43	50	15,35
Roza and Shizgal^27^	1584.80 ± 264.34	0.04	0.74	0.545	0.541	271.34	51	14,35
FAO.WHO.UNU, weight^28^	1584.70 ± 279.41	0.04	0.73	0.534	0.531	274.51	46	16,38
FAO.WHO.UNU, weight and height^28^	1606.20 ± 298.60	0.01	0.73	0.533	0.530	274.67	47	13,40
Schofield^3^	1572.75 ± 278.62	0.14	0.73	0.532	0.529	274.94	46	18,36
Owen et al^29^	1435.73 ± 251.78	< 0.001	0.74	0.551	0.548	269.4	39	41,20
Mifflin et al^15^	1493.20 ± 264.03	0.05	0.75	0.564	0.561	265.41	49	27,24
De Lorenzo et al^30^	1399.55 ± 318.61	< 0.001	0.75	0.568	0.564	264.4	39	49,12
Müller et al^31^	1523.44 ± 300.30	0.55	0.68	0.457	0.453	296.28	49	22,29
Livingston and Kohlstadt^32^	1505.43 ± 247.88	0.15	0.74	0.545	0.545	269.76	48	25,27
Ganpule et al^33^	1460.40 ± 266.80	0.001	0.75	0.561	0.550	266.35	40	38,29
Korth et al^34^	1644.45 ± 318.77	< 0.001	0.75	0.567	0.564	264.49	46	10,44
Frankenfield et al, without height^10^	1504.82 ± 253.89	0.14	0.74	0.555	0.552	268.05	51	22,27
Frankenfield et al, with height^10^	1579.92 ± 256.08	0.06	0.75	0.568	0.565	264.36	53	14,32
de la Cruz Marcos et al^35^	1708.71 ± 290.63	< 0.001	0.74	0.551	0.547	269.54	40	7,53
Nikooyeh et al^38^	1550.53 ± 279.02	0.60	0.72	0.524	0.521	277.36	50	19,31
Fairoosa et al^36^	1272.92 ± 257.05	< 0.001	0.72	0.510	0.514	279.07	28	68,4
Marra et al^37^	1564.07 ± 265.91	0.25	0.74	0.556	0.552	268.01	53	15,32
Nichols et al^39^	1464.23 ± 251.83	0.001	0.75	0.556	0.553	267.87	40	37,23
New equation	1524.25 ± 249.87	0.54	0.74	0.541	0.538	272.31	50	22,28

Abbreviations: REE; resting energy expenditure, PE; predictive equation, IC; indirect calorimetry, RAMSE; root mean square error, FAO; food and agriculture organization, WHO; world health organization, UNU; united nations university. Data are presented as mean ± standard deviation. Predicted REE is considered accurate when it is within ± 10% of measured REE. The percentage of subjects whose REE was predicted to be within ± 10% of measured REE was considered a measure of accuracy on an individual level. R2, adjusted R2, and RAMSE were estimated using linear regression. ^a^ Paired samples *t* test was used.

**Table 4:** Bland-Altman analysis for the agreement between REE-IC and REE-PEs in the validation group (n = 142)

**REE predictive equation**	**Mean difference (kcal/day),** **mean percentage error (%)**	**95% limits of agreement, kcal/day**	**Subjects outside limits of agreement, %**	**rho** ^a^
Harris and Benedict^12^	40, 6.0	-528.60 to 534.60	4.93	-0.49
Roza and Shizgal^27^	47, 6.6	-486.93 to 580.03	5.63	-0.52
FAO.WHO.UNU, weight^28^	46, 6.3	-490.30 to 583.21	4.22	-0.47
FAO.WHO.UNU, weight and height^28^	68, 7.5	-468.62 to 604.51	4.22	-0.40
Schofield^13^	34, 5.5	-503.13 to 572.13	4.22	-0.48
Owen et al^29^	-102, -3.6	-636.22 to 431.19	4.93	-0.58
Mifflin et al^15^	-45, 0.1	-568.43 to 478.34	5.63	-0.54
De Lorenzo et al^30^	-139, -7.0	-656.12 to 378.72	4.93	-0.33
Müller et al^31^	-15, 2.0	-596.34 to 478.34	7.04	-0.37
Livingston and Kohlstadt^32^	-33, 1.1	-568.43 to 502.79	5.63	-0.35
Ganpule et al^33^	-78, -2.2	-602.12 to 446.42	5.63	-0.53
Korth et al^34^	106, 9.9	-411.43 to 623.82	3.52	-0.33
Frankenfield et al, without height^10^	-33, 1.0	-564.24 to 497.38	5.63	-0.58
Frankenfield et al, with height^10^	42, 6.2	-482.39 to 565.72	5.63	-0.57
de la Cruz Marcos et al^35^	170, 14.7	-356.13 to 697.03	4.93	-0.44
Nikooyeh et al^38^	12, 4.1	-529.84 to 554.40	4.22	-0.47
Fairoosa et al^36^	-265, -14.9	-813.83 to 283.16	4.22	-0.55
Marra et al^37^	26, 5.0	-501.54 to 553.17	4.93	-0.53
Nichols et al^39^	-74, -1.7	-605.22 to 457.16	5.63	-0.59
New equation	-14, 2.4	-529.84 to 554.40	4.93	-0.59

Abbreviations: REE; resting energy expenditure, PE; predictive equation, IC; indirect calorimetry, FAO; food and agriculture organization, WHO; world health organization, UNU; united nations university. Mean difference = value of the difference between REE-PEs and REE-IC, mean percentage error = (REE-PEs-REE-IC/REE-IC) * 100 (a measure of accuracy on a group level). ^a^ Pearson’s correlation coefficients between the difference and average REE from IC and each predictive equation, indicating proportional bias. Significant (*P* < 0.05) rho are shown.

## Discussion

 We aimed to develop a new equation for the prediction of REE and to evaluate the validity of this equation and other REE-PEs in Tehranian adults. It is not new to develop an equation, as numerous studies have demonstrated that various REE equations can be applied to diverse populations.^13,34,39,40^ However, several studies found inaccuracies in the prediction of REE using commonly used predictive equations.^16,17,20^ Also, it is well established that these equations produce the best results when applied to people who have the same characteristics as those who developed them.^41^ This may be because an individual’s energy requirements may be influenced by race, as individuals of particular race may differ from each other in terms of their metabolic profile or anthropometric characteristics that affect metabolic characteristics.^42^ It seems that race affects REE significantly, as black subjects have lower REE than white subjects, regardless of potential confounding variables such as BMI or fat-free mass.^43^ Thus, due to the dependent effects of race on REE estimations, current equations should be adapted to account for race.

 Our findings showed that body weight, sex, and age were the main determinants of REE. Similar to previous research, there was a strong positive correlation between REE and age and gender; as well as a negative correlation with age.^12,44^ The mean REE predicted using this equation and the equations by Harris & Benedict, Schofield, Mifflin—St. Jeor, Müller, Livingston, Frankenfield, Nikooyeh, and Marra were not significantly different from REE measured by IC (mean bias = -45 to + 40 kcal/day). When assessing the bias at the group level, the new equation and the equations by Mifflin—St. Jeor, Livingston, Frankenfield, Nichols, Müller, and Ganpule demonstrated the best predictive values (bias of -2.2 to 2.4%). Regarding the accuracy at the individual level, the new equation (53% accuracy prediction) and the equations by Mara (53% accuracy prediction), Frankenfield (53% accuracy prediction), Roza (51% accuracy prediction), Nikooyeh (50% accuracy prediction), and Harris & Benedict (50% accuracy prediction) showed the greatest accuracies compared to the REE-IC. On one hand, applying the equations by Harris & Benedict, Roza, FAO/WHO/UNU, Schofield, and de la Cruz Marcos in our population showed the greatest overestimation of predicted REE compared to the REE-IC (more than 30%). In this case, our results are in accordance with the findings of some studies which concluded that the Harris-Benedict, FAO/WHO/UNU, and Schofield equations significantly overestimated REE compared to the REE-IC.^38,45,46^ In contrast, in other studies, the estimation of RMR using Harris-Benedict and FAO/WHO/UNU equations did not show considerable bias from the REE-IC.^37,47^ On the other hand, our findings showed the greatest underestimation of REE using the equation by Owen, De Lorenzo, Ganpule, Fairoosa, and Nichols (more than 35%). In contrast to ours, the results of a study showed significant overestimation by the De Lorenzo equation and a relatively good accuracy prediction by the Owen equation in a sample of Greek adults.^46^ As mentioned, for the new equation and all other equations evaluated, there was a considerable error in predicting REE at an individual level. Additionally, Bland Altman plots of all equations showed a lack of agreement with measured REE with wide limits of agreement. However, it appears REE was overpredicted at lower levels and underpredicted at higher levels, which is consistent with previous findings.^16,20,48-50^ Also, the findings at individual levels might be in part due to errors in REE measurement. These Errors may be because of an air leak, an inaccurate calibration of the calorimeter, involuntary periods of hyperventilation and hypoventilation, fluctuation in fractional-inspired oxygen concentration, or acid-base disturbances.^51^ Moreover, even though REE measurements were conducted under strictly standardized conditions, variations in eating and activity patterns in the days preceding REE measurements might also result in biological intra-individual variation in REE. It should be noted that the newly developed equation in our study analogous to the equation by Frankenfield which uses easily assessed characteristics (weight, age, and sex) may offer better estimates of REE in Tehranian adults compared to other previously published equations. These equations demonstrated relatively low mean percentage bias and adequate prediction accuracy, with approximately equal rates of overprediction and underprediction compared to other equations. Nevertheless, neither of these equations can be recommended for predicting individual REE in a clinical setting due to large individual errors.

## Strengths and limitations

 This study has some strengths. The study included subjects with wide age and BMI range from both sexes. Also, we reviewed the available formulas and compared them with our newly developed formula and the gold standard, IC. This study had also some limitations. First, body composition (fat mass and fat-free mass) was not considered in the equation. We made this decision since we believed body composition would not be incorporated into daily clinical practice. Second, despite recruiting the participants from several districts surrounding Tehran City, the new equation may not represent the entire Iranian population. Third, the study was conducted on subjects without diseases or comorbidities, so interpretations of the findings should be made with caution if they are generalized to persons with diseases or comorbidities. Fourth, although the newly developed equation could predict REE at the group level, its application at the individual level is questionable. Therefore, in individuals where a precise determination of REE is indicated (such as athletes), measurement by IC instead of the prediction of REE using equations is highly recommended.

## Conclusion

 In conclusion, this study highlights the importance of considering race when predicting REE and demonstrates that the newly developed equation for this population of Tehranian adults is more appropriate for predicting REE in a clinical setting at the group level. However, enthusiasm for recommending this equation for predicting individual REE in a clinical setting is damped by the large individual error evident with the equation.

## Acknowledgments

 The authors would like to thank the participants for their kind cooperation.

## Competing interests

 The authors declare no competing interests.

## Ethics Approval

 The study was approved by the ethics committee of Tehran University of Medical Sciences (Ethics number: IR.TUMS.MEDICINE.REC.1399.1294). Written informed consent was obtained from all patients.
